# Completeness of Reporting of Chronic Hepatitis B and C Virus Infections — Michigan, 1995–2008

**Published:** 2013-02-15

**Authors:** Kim Kirkey, Karen MacMaster, Anil Suryaprasad, Fujie Xu, Monina Klevens, Henry Roberts, Anne Moorman, Scott Holmberg, Jenna Webeck

**Affiliations:** Michigan Dept of Community Health; Div of Viral Hepatitis, National Center for HIV/AIDS, Viral Hepatitis, STD, and TB Prevention; EIS officer, CDC

Chronic hepatitis B virus (HBV) and chronic hepatitis C virus (HCV) infections are leading causes of death from cirrhosis and hepatocellular carcinoma in the United States (1). Because underreporting has complicated the understanding of disease burden, in 2010 the Institute of Medicine requested that CDC perform a comprehensive evaluation of national viral hepatitis surveillance (1). Hepatitis surveillance data rely on local and state estimates, and a better understanding of reporting at these levels can inform strategies to improve national data quality. As an initial assessment, CDC partnered with the Michigan Department of Community Health (MDCH) and an urban health-care system in southeastern Michigan to evaluate the completeness of reporting (including case status, demographic, and risk factor information) of cases of chronic HBV and HCV infection among persons who were enrolled in a multicenter chronic hepatitis cohort study (2) to the MDCH viral hepatitis registry. This report summarizes the results of that assessment. Among clinically confirmed chronic hepatitis infections, 82% of HBV infections and 65% of HCV infections were reported. Completeness of reporting of chronic HBV and HCV infections was significantly improved for those with more recent clinical diagnoses, but reporting still remained incomplete. The completeness of reporting varied significantly by demographic characteristics of patients with HCV infection. Few reports of either HBV or HCV infection included risk factors. Improving surveillance of chronic hepatitis in Michigan will require exploration of more efficient methods for the transfer of laboratory and clinical data and evaluation of the most appropriate sources for risk factor information to aid in the prevention of viral hepatitis transmission. Similar collaborations with health-care institutions that use electronic *International Classification of Diseases, Ninth Revision* (ICD-9) codes and laboratory data can provide local and state health departments with insight into the challenges to case reporting in their jurisdictions.

Reporting of chronic HBV and HCV infections became mandatory in Michigan in 2004 and 2000, respectively. In 2004, electronic dual reporting of both infections by laboratories and by health-care providers began with the launch of the Michigan Disease Surveillance System (MDSS). As part of an ongoing, multicenter, chronic hepatitis cohort study, investigators compiled clinical data from patients suspected to have chronic HBV or chronic HCV infection at any time during 2006–2008 and who resided in Michigan and sought care within the health-care network, which was comprised of several hospitals and clinics serving approximately 1 million Michigan residents (2). As of 2011, this health-care network had been documented by MDCH as a reporting institution for approximately 8% of all state reports of chronic HBV infection and 6% of all reports of chronic HCV infection since the advent of MDSS. According to U.S. Census data, the health-care network’s patient population has a higher representation of blacks (37% versus 25%), females (57% versus 51%), persons aged ≥65 years (20% versus 13%), and persons aged <17 years (24% versus 20%) than the surrounding regional population.

Clinically confirmed cases of chronic HBV and chronic HCV infection were identified in the cohort study by methods that have been described previously (2) (Box). To evaluate the completeness of reporting of chronic HBV and HCV infections, all clinically confirmed cohort cases found in the health-care system during the study period of 2006–2008 were matched to cases reported to MDSS by first name, last name, and date of birth, using probabilistic record-linkage software. The year of initial diagnosis (i.e., the year of the first written diagnosis or laboratory evidence of infection) among cohort cases found in the health-care system ranged from 1995 to 2008.

Cases from the cohort study that matched in MDSS were queried for case classification in MDSS (acute, chronic, or both) and for the presence of age, sex, and risk factor data in MDSS. For cohort patients coinfected with HBV and HCV who met confirmation criteria for only one infection, only the diagnosis meeting definitive inclusion criteria was considered a case. Age, sex, and year of initial diagnosis were examined for their association with completeness of reporting. Differences between the proportions of confirmed cases reported, by age, sex, and race/ethnicity, were tested for statistical significance by chi-square test. Year-to-year differences in the proportions of cases reported were assessed for trend using the Cochrane-Armitage trend test and year of initial diagnosis.

In the cohort of 4,393 persons, 14% had HBV infections, 85% had HCV infections, and 1% had coinfections, yielding a total of 670 HBV and 3,796 HCV infection cases. Of the HBV infection cases, 597 (89%) met clinical confirmation criteria for chronic HBV infection (29 by physician diagnosis alone and 568 by laboratory criteria with or without a physician diagnosis). Of the HCV infection cases, 3,036 (80%) met clinical confirmation criteria for chronic HCV infection (115 by physician diagnosis alone and 2,921 by laboratory criteria with or without a physician diagnosis). A total of 490 (82%) of the 597 confirmed cases with chronic HBV infection were matched to MDSS, and 1,967 (65%) of the 3,037 confirmed cases with chronic HCV infection were matched. Of the cases matched to MDSS, sex was reported in 99.6% (488 of 490) of HBV infection cases and 99.0% (1,947 of 1,967) of HCV cases. Race/ethnicity was reported for 75.1% and 66.7% of cases, respectively. Risk factor data were reported for <5% of HCV infection cases because of inadequate health department resources for case follow-up. HBV infection risk factor data were not recorded because of the absence of risk factor–related questions in case questionnaires. Of the 597 chronic HBV infections, 463 (78%) were appropriately classified as chronic in MDSS. Of the 3,036 chronic HCV infections, 1,918 (64%) were appropriately classified as chronic in MDSS (Table 1).

BOX **Criteria for identifying clinically confirmed cases of HBV and HCV infection in a cohort study — Michigan, 2006–2008**

**Confirmed chronic HBV infection if either of the following criteria are met** *

**Criteria 1**

**Specialist and primary-care provider documentation criteria**

Written/dictated description in a progress note by a specialist (hepatologist, gastroenterologist, or infectious disease specialist) or patient’s primary-care provider† that describes patient as having chronic HBV infection, being a HBV carrier, or having nonreplicating HBV.

**Criteria 2**

**Laboratory criteria**

Any two of the following test results at least 6 months apart: HBsAg positive, HBV DNA positive, or HBeAg positive. (Any combination of these tests performed ≥6 months apart is acceptable.)

**Confirmed chronic HCV infection if either of the following criteria are met** §

**Criteria 1**

**Specialist and primary-care provider documentation criteria**

Written/dictated description in a progress note by a specialist (hepatologist, gastroenterologist, or infectious disease specialist) or patient’s primary-care provider† that describes patient as having chronic HCV infection.

**Criteria 2**

**Laboratory criteria**

Has any of the following test results:

- Anti-HCV (hepatitis C antibody) positive by enzyme immunoassay (EIA or ELISA)
- HCV RIBA (recombinant immunoblot assay) positive
- HCV RNA detectable
- Report of HCV genotype

**AND** followed ≥6 months later by either of the following:

- HCV RNA detectable
- Report of HCV genotype

**Criteria 3**

**Combination clinical and laboratory criteria**

Patient has not presented with acute hepatitis (a discrete onset of any sign or symptom consistent with acute viral hepatitis [e.g., anorexia, abdominal discomfort, nausea, or vomiting, and either 1) jaundice or dark urine, or 2) serum ALT levels >400 IU/L])

**AND** has either of the following:

- HCV RNA detectable
- Report of HCV genotype

**Source:** Moorman AC, Gordon SC, Rupp LB, et al. Baseline characteristics and mortality among people in care for chronic viral hepatitis: the chronic hepatitis cohort study. Clin Infect Dis 2013;56:40–50.

**Abbreviations:** HBV = hepatitis B virus; HCV = hepatitis C virus; HBsAg = hepatitis B surface antigen; HBeAg = hepatitis B e antigen; ICD-9 = *International Classification of Diseases, Ninth Revision*; EIA = enzyme immunoassay; ELISA = enzyme-linked immunosorbent assay; RIBA = recombinant immunoblot assay; ALT = alanine aminotransferase.

Completeness of reporting of chronic HBV and HCV infection consistently improved over time and varied significantly by the year of diagnosis, with more complete reporting among cases with more recent diagnoses (p<0.001). Reporting of confirmed cases of HCV infection varied significantly by age group (p=0.001), sex (p=0.049), and race/ethnicity (p=0.024); reporting of these cases was more complete among persons aged 0–30 years, among males, and among non-Hispanic whites and Asians/Pacific Islanders (Table 2).

## Editorial Note

This initial evaluation of viral hepatitis surveillance in Michigan showed that reporting of chronic HBV and HCV infections was incomplete. However, reporting has improved over time, with more recently diagnosed cases significantly more likely to be reported and included in state surveillance data, particularly after dual reporting by laboratories and health-care providers began in 2004. Incomplete reporting and demographic disparities in reporting of chronic HCV infections should be considered when using surveillance data to estimate actual disease burden. Information on risk factors for infection, which could inform prevention efforts, were seldom reported because of the constrained resources for case follow-up of HCV infections and the absence of risk factor–related questions in HBV case forms.

Case reporting of notifiable infectious conditions is intended to describe disease burden, facilitate case management, ascertain risk factors to prevent transmission, identify and curtail outbreaks, and monitor implementation and impact of public health recommendations (3). Several challenges complicate chronic hepatitis surveillance efforts. The number of cases eligible for reporting is large, and data management is burdened by the reporting of numerous laboratory tests meeting the case definition per individual case (4). A previous comprehensive evaluation of viral hepatitis surveillance programs underscored the need for additional resources to achieve better investigation and case management of reported chronic viral hepatitis infections (5). Because of the challenges of case reporting, few states reported chronic HBV cases (11 through passive surveillance and eight through active surveillance) and chronic HCV cases (eight through passive surveillance and eight through active surveillance) to CDC in 2010 (6).

Chronic viral hepatitis cases might not be reported for several reasons. First, many cases, particularly before 2008, were reported to health departments by fax, which has made the completeness subject to the limitations of manual entry of cases by health departments. Second, older cases might have been diagnosed before mandatory reporting was implemented. Third, cases are often diagnosed at outside institutions and referred to the health-care system; in the transfer of care, case reports might not have been made by the diagnosing institution.

What is already known on this topic?

There are many challenges to surveillance of chronic hepatitis B virus (HBV) and chronic hepatitis C virus (HCV) infections, including the large number of cases eligible for reporting and the multiple laboratory tests required for each identified case. In 2010, the Institute of Medicine requested that CDC perform a comprehensive evaluation of the national viral hepatitis surveillance system.

What is added by this report?

In partnership with the Michigan Department of Community Health (MDCH) and an urban health-care system in southeastern Michigan, CDC evaluated the completeness of reporting of cases of chronic HBV and HCV infection among persons enrolled in a chronic hepatitis cohort study to MDCH’s viral hepatitis registry. Overall, 82% of chronic HBV infection cases and 65% of chronic HCV infection cases were reported; recently diagnosed cases were more likely to be reported. Basic demographic data were included for most reported cases, but risk factor data rarely were reported.

What are the implications for public health practice?

In Michigan, reporting of chronic viral hepatitis is improving since the adoption of a dual laboratory and health-care provider-based electronic reporting system, but still remains incomplete. As a specific response to the Institute of Medicine report and action plan, this evaluation serves as a model for evaluating viral hepatitis surveillance in other states. Improving surveillance of chronic hepatitis will require electronic transfer of laboratory and clinical data and alternate sources to obtain other information, such as risk factor data, necessary for prevention and case management.

The findings in this report are subject to at least four limitations. First, the matching of actual cases with MDSS is subject to potential misclassification of clinically confirmed cases by study investigators. Second, detection of cases in MDSS is subject to the limitations of matching, and at least some cases might be missed by changes in names or changes in residence. Third, reporting at the participating health-care system’s facilities might not be representative of reporting at other clinical-care or testing centers. Finally, in some cases, the year of diagnosis might be different from the year of the report, so the observed trends in reporting should be interpreted with caution.

This evaluation was possible because the state health department and hospital officials were willing to take a critical look at reporting for purposes of quality improvement. As an important response to the Institute of Medicine report and action plan (1), this evaluation serves as a model for similar efforts in other states and, in fact, will be replicated in other states with facilities participating in the cohort study.

The improvements in reporting of chronic HBV and HCV infections in Michigan coincide with improvements statewide in automated laboratory reporting, and a more detailed investigation of the association between the two factors is warranted. The persisting gaps in reporting highlight the need for more efficient means of transferring and interpreting reportable data. In previous studies, electronic reporting has been shown to improve the reporting of notifiable diseases, including hepatitis (7–8), and might be a method for improving the quality of reporting. For example, investigators at the U.S. Department of Veterans Affairs found that ICD-9 codes for HCV infections were highly predictive of actual HCV infection in their administrative databases (9).

Given the complexity of chronic hepatitis surveillance and the limited resources available, public health authorities should explore new strategies to improve reporting, such as wider adoption of electronic reporting. This report offers a roadmap for using large datasets from clinical institutions to provide state and local health departments with insight into the disease burden represented by chronic viral hepatitis case reports. The findings suggest the need for exploration of additional data sources for risk factor information, especially because data in chronic viral hepatitis case reports might not reflect the current risk for secondary transmission. Such a critical evaluation of surveillance data can help inform efforts to improve linkages to care and to prevent viral hepatitis transmission.

## Figures and Tables

**TABLE 1:** Completeness of reporting for clinically confirmed cases of HBV and HCV infection,* and corresponding case classification of the reported cases in the Michigan Disease Surveillance System — Michigan, 1995–2008

Clinical classification	Reported cases and classification								Unreported	
	
	Total		Acute		Chronic		Both		Total	
				
	No.	(%)	No.	(%)	No.	(%)	No.	(%)	No.	(%)
Confirmed HBV	**490/597**	**(82)**	27/597	(6)	400/597	(67)	63/597	(11)	**107/597**	**(18)**
Confirmed HCV	**1,967/3,036**	**(65)**	49/3,036	(2)	1,870/3,036	(62)	48/3,036	(2)	**1,069/3,036**	**(35)**

**Abbreviations:** HBV = hepatitis B virus; HCV = hepatitis C virus.

* Confirmed cases were considered to be cases identified in the cohort study by published methods (Moorman AC, Gordon SC, Rupp LB, et al. Baseline characteristics and mortality among people in care for chronic viral hepatitis: the chronic hepatitis cohort study. Clin Infect Dis 2013;56:40–50). Cases were confirmed by a combination of written diagnoses by health-care providers, *International Classification of Diseases, Ninth Revision* coding, and laboratory data consistent with a chronic HBV and a chronic HCV diagnosis. Reported cases were considered to be the clinically confirmed cases that were successfully matched to and identified in the Michigan Diseases Surveillance System.

**TABLE 2:** Completeness of reporting for confirmed cases of chronic HBV and HCV infection,* by selected characteristics — Michigan, 1995–2008

	Confirmed HBV (N = 597)			Confirmed HCV (N = 3,036)		
		
Characteristic	No. reported (n = 490)	(%)	p-value	No. reported (n = 1,967)	(%)	p-value
**Age group (yrs)**			**0.419**			**0.001**
0–30	66/85	(78)		60/80	(75)	
31–44	201/241	(83)		303/486	(62)	
45–54	128/150	(85)		989/1489	(66)	
55–64	64/80	(80)		481/735	(65)	
≥65	31/41	(76)		134/246	(54)	
**Sex**			**0.963**			**0.049**
Female	182/222	(82)		727/1161	(63)	
Male	308/375	(82)		1240/1875	(66)	
**Race/Ethnicity**			**0.548**			**0.024**
White, non-Hispanic	162/191	(85)		832/1242	(67)	
Hispanic	2/2	(100)		19/30	(63)	
Black, non-Hispanic	160/203	(79)		928/1496	(62)	
Asian/Pacific Islander	96/115	(83)		53/73	(73)	
Other/Unknown	70/86	(81)		135/195	(69)	
**Year of first diagnosis** †			**<0.001**			**<0.001**
1995–1996	41/65	(66)		99/178	(56)	
1997–1998	21/26	(78)		130/260	(50)	
1999–2000	25/34	(71)		159/321	(50)	
2001–2002	44/61	(73)		198/329	(60)	
2003–2004	53/65	(84)		221/380	(58)	
2005–2006	188/222	(84)		691/955	(72)	
2007–2008	118/123	(94)		469/613	(77)	

**Abbreviations:** HBV = hepatitis B virus; HCV = hepatitis C virus.

* Confirmed cases were considered to be cases identified in the cohort study by published methods (Moorman AC, Gordon SC, Rupp LB, et al. Baseline characteristics and mortality among people in care for chronic viral hepatitis: the chronic hepatitis cohort study. Clin Infect Dis 2013;56:40–50). Cases were confirmed by a combination of written diagnoses by health-care providers, *International Classification of Diseases, Ninth Revision* coding, and laboratory data consistent with a chronic HBV and a chronic HCV diagnosis. Reported cases were considered to be the clinically confirmed cases that were successfully matched to and identified in the Michigan Diseases Surveillance System.

† One case of chronic HBV infection from the cohort was missing data on year of first diagnosis.

## Footnotes

* Patients who are confirmed per these criteria to have had chronic HBV infection at any point, but who later cleared the disease (spontaneously or as a result of treatment), belong in the cohort and should be classified as having a confirmed case of HBV infection.

† This must be a textual description within a progress note, with or without an ICD-9 code. The primary-care provider should appear to have an informed, confident basis for the diagnosis based on serologic results and/or patient history, or the citation of outside laboratory studies that corroborate the diagnosis.

§ Patients who are confirmed via these criteria to have had chronic HCV infection, but who have been successfully treated and have cleared HCV RNA, belong in the cohort and should be classified as having a confirmed case of HCV infection.
